# P05.62. Clinical identification of patient propensities to choose integrative medicine for back pain: results from the SPICER project

**DOI:** 10.1186/1472-6882-12-S1-P422

**Published:** 2012-06-12

**Authors:** M Aickin, H Tick, A McCaffery, C Ritenbaugh, G Pugh, D Himick

**Affiliations:** 1 University of Arizona, Tucson, USA; 2 Marino Centers for Integrative Health, Boston, USA

## Purpose

Back pain is one of the major sources of disability in the US, but existing studies often have a restricted focus, leaving many patients unstudied.. The SPICER project is designed to assess the effect of integrative medical care in a mixed integrative/conventional clinic, using only existing electronic medical records, without any patient exclusions. This report will present information on which patient characteristics are good candidates to be used for adjustment or matching to reduce treatment selection bias.

## Methods

All diagnoses, visits, services, procedures, claims, and medications were extracted on 8,000 back pain patients from 2002 to 2009. Integrative medicine services were acupuncture, bodywork (including massage), and chiropractic. Event-stream data methods were used to construct analysis datasets containing potential matching factors: gender, age, year of first visit, comorbidities, combinations of 7 categories of back pain at first diagnosis, and use of integrative medicine prior to diagnosis of back pain.

## Results

The volume of results is considerable, because no patients were excluded for having a complex medical record. In general, selection of integrative medicine for back pain was positively related to being female, being older, having used it before back pain diagnosis (thus prior acupuncture use is strongly related to its later use, and similarly for the other treatments), strongly related to the complexity of the initial back pain diagnosis, and tending to decrease over calendar time.

## Conclusion

Identification of clinical variables that contribute to the propensity to use integrative medicine is vitally important for non-intervention research from medical records. All techniques for reducing treatment selection bias depend on them. The results reported here show that there is an adequate battery of matching factors that are in concert strongly related to choice of therapy.

